# "Cracked Nipples" and the Management of the Breasts

**Published:** 1908-04-11

**Authors:** 


### Obstetrics.

### "CRACKED NIPPLES" AND THE MANAGEMENT OF THE BREASTS.

Among the minor troubles of the puerperium, "cracked nipples" give rise to much suffering to the patient and annoyance to the practitioner. They may, too, be a source of very serious disturbance if they become infected, because lymphatic absorption from them is the one important cause of acute mastitis and mammary abscess. Practically every case of mammary abscess can be traced to a fissure in the nipple, sometimes so small as to have given rise to only passing trouble. They are commonly stated to be due to dirt, the wearing of stays, and improper preparation of the nipples for lactation. None of these causes, however, can be looked upon as carrying conviction, for dirt and the wearing of stays are common enough in women who have not babies, and yet we never see cracked nipples in them; and, again, it is by no means certain what is the best method of preparing the nipples for lactation, and it is probably often true that the very means adopted really dispose to fissure. As an example, applying brandy to the nipples is quite commonly recommended for a few weeks before lying-in. It is difficult to believe that this will do anything but harm by hardening the epithelium, making it brittle, and therefore more likely to crack. It practically comes to this, that the nipples, if well formed, require very little preparation for lactation, provided they are not scaly and are kept supple by the application occasionally of a little simple antiseptic ointment. Inasmuch, therefore, as women without babies practically never get cracked nipples, it must be admitted that it is the baby which causes the trouble. The reason for this is not far to seek. The baby is commonly put to the breast within twenty-four hours of delivery, when there is generally no free milk present, and then, getting nothing, his gums may rub off some of the epithelium and cause a fissure. Or, again, in some cases the breasts are tender, hard, and swollen at the end of the second or third day, and the milk does not run freely, and for the same reason as before the epithelium is rubbed off. The same thing happens when the nipples are badly formed and the baby cannot get hold of them easily. The way to prevent fissures, then, is to abstain from putting the baby to the breast until it is likely that there is milk being secreted, or at least to limit the time during which it should make its first efforts. If cracks have actually formed, the treatment should be on a purely antiseptic principle, like the treatment of any other wound. Glycerine of tannic acid is the most commonly used application, but who would think of treating a wound anywhere else with it? Balsam of Peru is used by some with more justification, but it is not an elegant preparation and is but a mild antiseptic. The method under which fissures heal best is to keep them constantly wet with an antiseptic lotion, preferably a non-poisonous one—those in commonest use being saturated solution of boric acid, and sulphurous acid diluted to half its strength with water. A very small pad soaked in either and covered by gutta percha tissue leads, as a rule, to healing in forty-eight hours. It is essential that the affected breast should be used in the meantime, so that it does not become greatly congested and therefore more liable to infection from the crack. As it is exceedingly painful to suckle from a cracked nipple, a glass nipple-shield should be used, and the nurse should be instructed to see that the flow of milk is started before putting the baby to it. If the affected breast is given double as long a rest as the sound one, healing will proceed quickly and no dangerous congestion will ensue. If the breast becomes hard and painful it is better to apply first a hot fomentation, and hot oil is better than hot water, followed by gentle massage with the palm of the hand anointed with vaseline, from the periphery of the breast towards the nipple. This is at first very painful, but if done gently and persevered with, very soon streams of milk will begin to flow from the nipple and the baby may then be put to it to finish the cure. Such a breast is hard because the blood-vessels around the acini are greatly congested, not because the ducts are blocked with milk. This method of treatment is better than any form of breast pump, and better than putting the baby to the breast in the first instance, because the milk will not flow without previous fomentation and massage. When lotions are used the nipples must be thoroughly washed before the baby is put to them, and it is wise to wash out the baby's mouth before suckling as well as afterwards.

It is often a matter of some difficulty to know what is the best way to deal with the breasts in cases where the infant is born dead or dies soon after birth. If left alone they will become congested, hard, and painful, and may easily give rise to serious trouble or, at the very least, to much suffering. The best treatment for such breasts is to keep the blood out of them as much as possible by tight bandaging—not supporting with a shoulder bandage, but actually compressing them against the ribs with a flannel bandage rolled round the chest. The breasts should be covered with a thin layer of cotton wool and then held up towards the mid-line in the non-dependent positions. Then using a thin flannel or domette bandage, commencing under one breast, passing over the other, round the back, over the first and under the second, a real figure-of-eight bandage is put on for several complete turns. The amount of pressure which can be borne without undue discomfort can only be found by trial. This method does not absolutely prevent blood getting to the breasts, but it limits the flow, so that secretion is delayed and very quickly ceases. Should the breasts become a little painful they may be massaged and then the bandage reapplied as firmly as can be tolerated. About five days of this treatment usually suffices to arrest milk-secretion.

